# Novel Insights into the Effect of *Pythium* Strains on Rapeseed Metabolism

**DOI:** 10.3390/microorganisms8101472

**Published:** 2020-09-25

**Authors:** Kateřina Bělonožníková, Kateřina Vaverová, Tomáš Vaněk, Miroslav Kolařík, Veronika Hýsková, Radomíra Vaňková, Petre Dobrev, Tomáš Křížek, Ondřej Hodek, Kateřina Čokrtová, Adam Štípek, Helena Ryšlavá

**Affiliations:** 1 Department of Biochemistry, Faculty of Science, Charles University, Hlavova 2030, 128 43 Prague 2, Czech Republic; katerina.belonoznikova@natur.cuni.cz (K.B.); katerina.vaverova@natur.cuni.cz (K.V.); veronika.hyskova@natur.cuni.cz (V.H.); 2 Biopreparáty, spol. s r.o., Tylišovská 1, 160 00 Prague 6, Czech Republic; vanek@biopreparaty.eu (T.V.); stipek@biopreparaty.eu (A.Š.); 3 Institute of Microbiology, Academy of Sciences of the Czech Republic, Vídeňská 1083, 142 20 Prague 4, Czech Republic; mkolarik@biomed.cas.cz; 4 Department of Botany, Faculty of Science, Charles University, Benátská 2, 128 01 Prague 2, Czech Republic; 5 Institute of Experimental Botany, Academy of Sciences of the Czech Republic, Rozvojová 263, 165 02 Prague 6, Czech Republic; Vankova@ueb.cas.cz (R.V.); Dobrev@ueb.cas.cz (P.D.); 6 Department of Analytical chemistry, Faculty of Science, Charles University, Hlavova 2030, 128 43 Prague 2, Czech Republic; tomas.krizek@natur.cuni.cz (T.K.); ondrej.hodek@natur.cuni.cz (O.H.); katerina.cokrtova@natur.cuni.cz (K.Č.)

**Keywords:** *Pythium*, secretome, cultivation medium, plant metabolism, phytohormones, rapeseed, enzyme activities

## Abstract

*Pythium oligandrum* is a unique biological control agent. This soil oomycete not only acts as a mycoparasite, but also interacts with plant roots and stimulates plant defense response via specific elicitors. In addition, *P. oligandrum* can synthetize auxin precursors and stimulate plant growth. We analyzed the secretomes and biochemical properties of eleven *Pythium* isolates to find a novel and effective strain with advantageous features for plants. Our results showed that even closely related *P. oligandrum* isolates significantly differ in the content of compounds secreted into the medium, and that all strains secrete proteins, amino acids, tryptamine, phenolics, and hydrolytic enzymes capable of degrading cell walls (endo-β-1,3-glucanase, chitinase, and cellulase), exoglycosidases (especially β-glucosidase), proteases, and phosphatases. The most different strain was identified as a not yet described *Pythium* species. The changes in metabolism of *Brassica napus* plants grown from seeds coated with the tested *Pythium* spp. were characterized. Enhanced levels of jasmonates, ethylene precursor, and salicylic acid may indicate better resistance to a wide variety of pathogens. Glucosinolates, as defense compounds against insects and herbivores, were enhanced in young plants. Altogether, *P. oligandrum* strains varied in their life strategies, and either they could perform equally as plant growth promoters and mycoparasites or they had developed one of these strategies better.

## 1. Introduction

Biological control agents (BCAs) represent a promising, environmentally friendly approach to protecting plants against a vast array of pathogens. These agents include fungi, oomycetes, and bacteria, and their positive effect stems from interactions that prime defense responses and enhance plant resistance to future stressors [1]. For example, soilborne plant pathogens can be biologically controlled by treating seeds with antagonists [2]. In particular, a striking number of soilborne diseases of various plants can be controlled by *Pythium oligandrum*, including ascomycetes, basidiomycetes, and pathogenic oomycetes [3].

*P. oligandrum* is a nonpathogenic soil inhabitant with worldwide distribution, which belongs to the class of filamentous eukaryotic microorganisms (oomycetes) and stands out among promising licensed biological agents. *P. oligandrum* has numerous beneficial effects on plant fitness, which result from the synergistic action of direct and indirect mechanisms on plant protection. *P. oligandrum* directly attacks the aforementioned pathogens via mycoparasitism or antibiosis, even attacking pathogenic oomycetes of its own *Pythium* genus [1,3,4,5]. Simultaneously, *P. oligandrum* also competes for nutrients and/or space with its target organisms.

*P. oligandrum* as a BCA can colonize plant root systems without damaging plant tissues, produce elicitors, secrete different hydrolases, and trigger signaling cascades and defense gene expression, thus indirectly activating localized and systemic induced resistance [3]. It produces two types of proteinaceous elicitors including the extracellular protein oligandrin (10.5 kDa) and cell wall protein fractions [6,7]. Oligandrins function like sterol carrier proteins and pick up sterols from plasma membranes, because oomycetes are unable to synthesize sterols but require an exogenous source of β-hydroxy sterols for sporulation [6].

Therefore, the treatment of plants with *P. oligandrum* functions by priming and activating the plant immune system, and thus providing increased protection against fungal and bacterial diseases [1,3]. In the rhizosphere, *P. oligandrum* supports the plant root system by producing tryptamine (auxin precursor), which is readily absorbed, thereby increasing the root mass through enhanced secondary root formation [3,8]. Since roots are highly sensitive to auxin, even at low concentrations, a mild but frequent exogenous auxin production may have a strong impact on root physiology [8].

The aim of this work was to find new *Pythium* strains exhibiting unique beneficial properties suitable for use as BCAs. For this purpose, we compared the activities of enzymes related to mycoparasitism or competition for nutrients (glycosidases, proteases, phosphatases, etc.), the amount of excreted oligandrins, and the amount of tryptamine as an auxin precursor and plant growth promoter. The secretomes of 11 *Pythium* spp. were analyzed in terms of protein profiles, total and individual free amino acids, and activity of both endo- and exoglycosidases, proteases, and phosphatases. In addition, we evaluated the effect on the metabolism of *Brassica napus* plants after seed coating. We found significant differences, especially in the contents of tryptamine, auxin, and other phytohormones, and free amino acids.

## 2. Materials and Methods

### 2.1. Isolation and Selection of Pythium Isolates

Approximately 20 g of soil sample was mixed thoroughly with 15 mL of sterilized (121 °C; 30 min) 15 g L^−1^ tap water agar (TWA) [9]. Then, it was poured into a Petri dish and left to solidify. After that, the surface of the Petri dish was covered with pure TWA, incubated in the dark at 24 °C, and observed daily.

After 5 days of incubation, the Petri dish with the soil sample was microscopically observed. In the case of ornamental oogonia detection, a small plug of agar medium with the oogonia was transferred to a Petri dish with TWA, followed by 24-h incubation at an elevated temperature (36–37 °C). After the incubation, a small plug of TWA with one separated growing hypha was transferred, using a sterile preparation needle and microscope, to a Petri dish with TWA and incubated for 24 h at 24 °C. After this last incubation period, a small plug of TWA with one separated hypha was transferred with a sterile needle to a Petri dish with malt extract agar (MEA, Merck, Darmstadt, Germany). After 7 days of incubation at 24 °C, the plugs of MEA with growing oomycete biomass were stored in paraffinic oil at 10 °C.

Only the strains with in vitro mycoparasitic abilities were selected for further study. The competition tests were conducted in triplicates on 9 cm diameter Petri dishes with MEA medium (25 °C, dark) using plant pathogenic fungi *Alternaria alternata*, *A. brassicicola*, *Verticillium albo-atrum*, and *Phoma lingam.* First, the plant pathogens were inoculated on one side of the plate and allowed to grow for 2–5 days, producing colonies 20–25 mm in size. Thereafter, the agar block with particular *Pythium* strain was placed onto the opposite side of the Petri dish, and the continuation of the growth of the tested mold and *Pythium* were evaluated every 2 days until 10 days. The ability of individual strain to parasitize the pathogens was assessed on the basis of visible overgrowth of the pathogen colony by *Pythium* hyphae, cessation of pathogen hyphae growth and loss or inability of the pathogen colony to change pigmentation compared to control growth without *Pythium* sp. Another characteristic feature of parasitism, which was evaluated using an optical microscope, was the ability of *Pythium* sp. hyphae to encircle the pathogen hyphae at sites of early contact between the two colonies.

### 2.2. Cultivation of Pythium Isolates

Eleven strains exhibiting spiny spores, a typical feature of *P. oligandrum*, and mycoparasitism ability were selected for the study. The composition of cultivation medium was 1000 g millet grain mixed with 500 mL of 2% (*w*/*v*) sucrose solution containing 30 μM cholesterol as a source of sterols. A quantity of 30 g of the solid cultivation medium was placed in a 250-mL Erlenmeyer flask, sterilized, inoculated by 5 small plugs of *Pythium* isolates, and incubated at 24 °C for 7 days. After the cultivation period, the biomass of growing mycelium and remnants of cultivation media were diluted 1:5 with sterile deionized water and centrifuged (8000× *g*, 15 min). The upper phase was used for the determination of secreted compounds. The biomass of *Pythium* was freeze-dried. The number of oospores was determined after lyophilization, and the inoculum concentration for further use (seed coating) was set to 12.0 × 10^6^ oospores.g^−1^ using sterile carrier silicon dioxide powder. Control (negative control) represents a sterile, non-inoculated cultivation medium cultivated under the same conditions. The M1 strain of *P. oligandrum* was provided by the company Biopreparáty, Ltd. (Únětice, Czech Republic) and corresponds to strain ATCC 38472. This oomycete was isolated from sugar beet [10].

### 2.3. Identification of Pythium Isolates

Partial sequences of cytochrome c oxidase subunit I (COI), which is recommended as the main marker for oomycete identification [11,12], were studied in all strains. In addition, internal transcribed spacer (ITS) rDNA, another recommended barcode [12], was sequenced in the strains representing unique COI haplotypes. Genomic DNA was extracted using a DNeasy UltraClean Microbial Kit (Qiagen, Germantown, MD, USA). The ITS region was amplified with the primers ITS4 and ITS5 [13]. The COI gene was amplified with the primers Fm85mod and OomCoxI-Levup [12]. Amplification conditions were set as in [14]. The PCR products were purified and sequenced in Macrogen, Europe. The dataset for the phylogenetic analyses was assembled using reference sequences generated by [12,14] and supplemented with best Blast hits from NCBI GenBank. The other two *P. oligandrum* sequences were extracted from the whole genomic data of ATCC 38472 [15] and Po37 [16]. Sequence alignments was performed in MAFFT 6 using the G-INS-i strategy [17]. Maximum Likelihood phylogenetic analyses were done in PhyML 3.1. [18] using 500 bootstrap replicates and default settings. The COI gene alignment had 28 sequences and 680 characters, of which 91 were variable and 46 were parsimony informative. The ITS alignment had 29 sequences and 755 positions, of which 24 were variable and 12 parsimony informative. Barcode sequences were deposited in the EMBL sequence database (Table 1).

### 2.4. Plant Material

*Brassica napus* (rapeseed) seeds were treated with lyophilized *Pythium* spp. inoculum (oomycete biomass with medium inoculum) containing 12.0 × 10^6^ oospores.g^−1^. The inoculation of seeds was performed in a rotating seed machine at the concentration of 5 g of the inoculum on 1 kg of seeds. The mixture was moistened with deionized water and exposed to continuous rotation for 5 min. Then, it was left to dry and stored at room temperature.

*Brassica napus* treated and nontreated seeds were sown in pots with soil and grown for three weeks in growth chambers under 16/8 h light/dark period, ca. 150 µmol (photon) m^−2^ s^−1^ irradiance, at 20 °C, and 60% relative humidity. The samples of all mixed leaves from each group were immediately frozen in liquid N_2_ and kept at −80 °C.

Frozen leaf samples (0.5 g) were ground in liquid N_2_ with 1% polyvinylpyrrolidone. Then, 0.5 mL of extraction buffer (0.1 M Tris–HCl, pH 7.8, 1 mM EDTA, 10 mM DTT, 5 mM MgCl_2_; except for glycosidases and proteases—100 mM MOPS-NaOH, pH 5) was added to the ground tissue. The homogenate was centrifuged at 13,000× *g* for 10 min, and the resulting supernatant was used for measurements.

### 2.5. Protein Content

Protein measurements of all samples were performed spectrophotometrically at 595 nm using Protein Assay solution (Bio-Rad, Hercules, CA, USA) with bovine serum albumin as a standard [19].

### 2.6. Electrophoretic Separation

Tricine-SDS-electrophoresis was performed to detect small molecules (such as oligandrins) secreted into the medium [20]. The gels consisted of 4% focusing and 16% separating layers. Medium samples were dissolved 1:1 in reducing sample buffer (6% (*w*/*v*) SDS; 3% (*v*/*v*) mercaptoethanol; 15% (*w*/*v*) glycerol; 0.025% (*w*/*v*) Coomassie Blue G-250; 150 mM Tris-HCl, pH 7.0). The proteins contained in the medium were separated by an SDS-PAGE run in 4% focusing and 15% separating gels [21].

### 2.7. Free Amino Acids and Tryptamine Content

Free proteinogenic amino acids and tryptamine were determined by capillary electrophoresis [22]. All electrophoretic experiments were conducted in a fused-silica capillary (Polymicro Technologies, Phoenix, AZ, USA) using a G7100A Capillary Electrophoresis Instrument (Agilent Technologies, Waldbronn, Germany) with a contactless conductivity detector. The detector consisted of two 4-mm-long cylindrical electrodes, with a 1-mm insulation gap. The inner diameter of the electrodes was 400 μm. The detection limits of all amino acids were in the range of 6 μg g^−1^ to 33 μg g^−1^. The limit of detection was calculated as 3-fold the standard deviation of the noise.

### 2.8. Phenolics and Antioxidant Capacity

The total phenolic content was determined using the standard Folin-Ciocâlteu colorimetric method [23,24]. Free Radical Scavenging, with the 2,2-diphenyl-1-picrylhydrazyl (DPPH) radical, and the Ferric Reducing Antioxidant Potential (FRAP) assays were used to determine the antioxidant capacity [23,24].

### 2.9. Phytohormone Analysis

In rapeseed leaves, indole-3-acetic acid (IAA), IAA-aspartate (IAA-Asp), indole-3-acetamide (IAM,), indole-3-acetonitrile (IAN), oxo-IAA (OxIAA), oxo-IAA-glucose ester (OxIAA-GE), phenylacetic acid (PAA), salicylic acid (SA), jasmonic acid (JA), JA-isoleucine (JA-Ile), and cytokinins (CKs) were determined. Frozen samples (ca. 50 mg fresh weight (FW)) were homogenized and extracted with cold (−20 °C) methanol/water/formic acid (15/4/1, *v*/*v*/*v*), as described previously [25,26,27]. The following isotope-labeled internal standards (10 pmol/sample) were added: ^13^C_6_-IAA (Cambridge Isotope Laboratories); 2H4-SA (Sigma-Aldrich); ^2^H_3_-PA, ^2^H_3_-DPA (NRC-PBI); ^2^H6-ABA, ^2^H_5_-JA, ^2^H_5_-transZ, ^2^H_5_-transZR, ^2^H_5_-transZ7G, ^2^H_5_-transZ9G, ^2^H_5_-transZOG, ^2^H_5_-transZROG, ^2^H_5_-transZRMP, ^2^H_3_-DZ, ^2^H_3_-DZR, ^2^H_3_-DZ9G, ^2^H_6_-iP, ^2^H_6_-iPR, ^2^H_6_-iP7G, ^2^H_6_-iP9G, ^2^H6-iPRMP (Olchemim). Phytohormones were separated with a reverse-phase cation exchange SPE column (Oasis-MCX, Waters) into the acid fraction by elution with methanol (auxins, ABA, SA, JA), and into the basic fraction by elution with 0.35 M NH_4_OH in 60% methanol (CKs and aminocyclopropane-1-carboxylic acid (ACC)). Fractions were analyzed using HPLC (Ultimate 3000, Dionex, Sunnyvale, CA, USA) coupled to a 3200 Q TRAP hybrid triple quadrupole/linear ion trap mass spectrometer (Applied Biosystems, Waltham, MA, USA). Hormone quantification was performed using the isotope dilution method with multilevel calibration curves (r^2^ > 0.99). Data processing was performed with the Analyst 1.5 software package (Applied Biosystems).

### 2.10. Extraction and Separation of Glucosinolates

Glucosinolates were extracted from 100 mg of lyophilized leaves ground in 1500 µL methanol/water (70/30, *v*/*v*). Then, the sample was shaken for 10 min (Vibramax 100 vibration platform shaker, Heidolph, Schwabach, Germany) and sonicated for 10 min (Elmasonic S 15, Elma, Germany). After centrifugation (10 min at 9000 rpm), 1200 µL of supernatant was evaporated and dissolved in 200 µL of background electrolyte (100 mM sodium tetraborate dissolved in water) and filtered through a 0.45-µm PVDF filter [28]. Again, the sample was shaken for 10 min and sonicated for 10 min. After centrifugation, 30 µL of supernatant was mixed with 5 µL of 1.8 mM sorbic acid as an internal standard.

Electrophoretic experiments were performed on an Agilent 7100 CE system (Agilent Technologies, Waldbronn, Germany) equipped with a UV/VIS detector. An unmodified fused-silica capillary of 50 µm ID and 375 µm OD (Polymicro Technologies, Phoenix, AZ, USA) was cut to 70.0 cm total length (effective length of 61.5 cm). Before the first measurement of the day, the capillary was flushed for 10 min with 1M NaOH and 10 min with deionized water. Between individual runs, the capillary was flushed with 1M NaOH for 2 min, and with background electrolyte for 2 min as well. Samples were injected using 5 kPa pressure for 5 s. Voltage of 20 kV (current approx. 76 µA) was applied during the 45-min analysis, and absorbance was detected at λ = 225 nm. The capillary temperature was 25 °C. The detection limits of glucosinolates were from 1 μg g^−1^ to 2 μg g^−1^. The limit of detection was calculated as 3-fold the standard deviation of the noise.

### 2.11. Enzyme Activities

Generally, enzyme activities were determined spectrophotometrically (Helios α, Thermo-Spectronics, Waltham, MA, USA) and expressed as activity in μmol of the respective product formed (substrate decreased) per min per mL of growth medium or per g of fresh plant weight (FW).

Catalase (CAT, EC 1.11.1.6) was detected at 240 nm as a H_2_O_2_ decomposition rate. Total ascorbate peroxidase (APOD, EC1.11.1.11) activity was determined as a decrease in the absorbance of ascorbate at 298 nm. Malic enzyme (NADP-ME, EC 1.1.1.40), glucose-6-phosphate dehydrogenase (EC 1.1.1.49), shikimate dehydrogenase (EC 1.1.1.25), and glutathione reductase (GR, EC 1.8.1.7) activities were assayed as NADPH production at 340 nm [29,30].

Proteolytic activity was determined using azocasein substrate or Nα-benzoyl-DL-arginine 4-nitroanilide [31,32]. Endo-β-1,3-glucanase (EC 3.2.1.6), cellulase (EC 3.2.1.4), and chitinase (EC 3.2.1.14) activities were determined based on the quantification of reducing sugars (products) with 3-methyl-2-benzothiazolinone hydrazone [33].

α-Glucosidase (EC 3.2.1.20), β-glucosidase (EC 3.2.1.21), α-galactosidase (EC 3.2.1.22), β-galactosidase (EC 3.2.1.23), α-mannosidase (EC 3.2.1.24), and β-N-acetylhexosaminidase (EC 3.2.1.52) activities were determined using the end-point method with specific synthetic *p*-nitrophenyl (*p*-NP-) substrates (*p*-NP-α,D-glucopyranoside, *p*-NP-β,D-glucopyranoside, *p*-NP-α,D-galactopyranoside, *p*-NP-β,D-galactopyranoside, *p*-NP-α,D-mannopyranoside, and *p*-NP-N-acetyl-β-D-glucosaminide, respectively). The reaction mixture contained a 50 mM citrate buffer, pH 4.5, 0.5 mM *p*-NP-substrate, and 10 µL of enzyme extract in total 40 µL volume. The reaction was stopped with 80 µL of 0.25 M borate buffer, pH 9.0. The hydrolyzed *p*-nitrophenol in the alkali region was quantified by measuring absorbance at 405 nm against a blank in which the enzyme was previously inactivated by the high pH of the borate buffer. Acid (EC 3.1.3.2) and alkaline phosphatase (EC 3.1.3.1) activities were determined with *p*-NP-phosphate at pH 5.5 and 9.0, respectively, modified from [34].

Superoxide dismutase (SOD, EC 1.15.1.1) isozyme patterns and activities were assessed after separation by 10% native PAGE [35,36].

Peroxidase (POD, EC 1.11.1.7) isozyme pattern was assessed after separation by 10% native PAGE. POD isozymes were detected in situ by staining gels in 1 M acetate buffer, pH 4.6, with 0.04% benzidine and 10 mM H_2_O_2_ [37].

Shikimate dehydrogenase (SDH, EC 1.1.1.25) isoenzyme pattern was detected after separation by 10% native PAGE by staining in 3 mM shikimate, 0.2 mM NADP^+^, 0.1 mg mL^−1^ nitroblue tetrazolium, and 5 µg mL^−1^ phenazine methosulfate.

### 2.12. Carbon, Nitrogen, and Sulfur Content Analysis

The lyophilized samples of leaves were used for elementary analysis of C, H, N, and S, which was performed on a Thermo Finningan Flash FA 1112 series CHNS/O analyzer.

### 2.13. Statistical Analysis

All measurements were performed at least in triplicate. Each plant experimental group consisted of three biological samples. Each sample represented mixed leaves of 30 plants. Three biological replications (i.e., independently cultured strains) were studied for each strain. Data were analyzed by one-way ANOVA (Bonferroni method) and *t*-test; differences were considered significant at *p* ≤ 0.05. The different letters in figures and tables denote significant differences (*p* ≤ 0.05).

## 3. Results and Discussion

### 3.1. Identification of Tested Pythium spp. Revealed not yet Described Pythium Species

**Figure 1:**
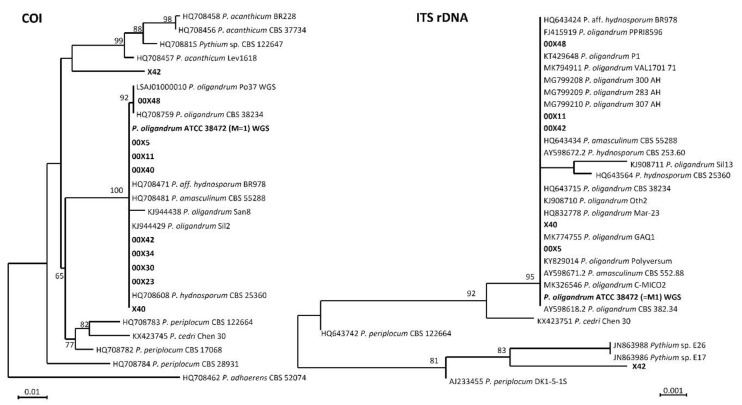
Maximum likelihood (ML) tree of *Pythium* spp. based on COI (left) and ITS rDNA (right) barcode genes. The bootstrap values are indicated at the nodes. The strains used in this study are printed in bold. The tree was rooted with *P. adherens* HQ708462 and *P. periplocum* AJ233455, respectively. Each code (00X5, 00X11, 00X23, 00X30, 00X34, 00X42, 00X48, X40, X42, and M1) represents a *Pythium* strain.

In response to demands from many regulatory authorities and professional agricultural institutes, scientists have intensified the search for new and more effective biological control agents, which requires characterizing not only the specific organisms but also their effects on plant metabolism. As *Pythium oligandrum* is well-known for its beneficial features, we searched for other *Pythium* oomycetes with high potential for use as BCAs. For this purpose, we collected soil samples from different areas to identify new suitable organisms with unique properties (Table 1, Figure 1). We selected the strains that after cultivation showed ornamental oogonia and also behaved as mycoparasites. The mycoparasitism tests of the M1 strain as an example are shown in the Supplementary Materials (Figures S1–S4). Then, we characterized unknown *Pythium* strains by COI and ITS rDNA barcode genes.

Based on the COI gene, we determined that our study strains belonged to three unique haplotypes: (1) X42, (2) 00X48, and (3) others. Phylogenetically, the two latter haplotypes belonged to the well-supported clade consisting of sequences deposited as *P*. *amasculinum* (HQ708481, [12]); *Pythium* aff. *hydnosporum* (HQ708471, [12]); *Pythium hydnosporum* (HQ708608, [12]); and *P. oligandrum* (HQ708759, [12]; KJ944429, [14]) (genomic data from strains Po37 and ATCC 38,472 = M1) (Figure 1). The sequence of strain X42 showed a separate position, with the closest affinity to sequences deposited as *Pythium acanthicum.* Based on the ITS, all but one (X42) of the strains belonged to a single haplotype. The main haplotype belonged phylogenetically to the well-supported clade of *P. oligandrum*, *P. hydnosporum*, and *P. amasculinum*, whereas strain X42 showed a separate position with the closest affinity to *Pythium* sp. E26 JN863988 (97.50% sequence similarity).

Most of our isolates fit into the *P. oligandrum* clade consisting of *P. oligandrum*, *P. hydnosporum*, and *P. amasculinum* sequences. All three species have spiny oogonia and are morphologically related. For these reasons, *P. oligandrum* has been often misidentified as *P. hydnosporum. P. amasculinum* is considered a likely synonym of *P. oligandrum* [38].

There is a general consensus that the species inside this clade cannot be recognized based on the widely used barcode genes ITS and COI and should be formally synonymized pending more thorough investigations with multiple hypervariable genes [11,12,14]. Our strains morphologically fit into *P. oligandrum*, and accordingly we are designating them using this name. The strain of X42 has a separate position, sister to the *P. oligandrum* clade and *P. acanthium*. This strain is not ascribable to any sequenced species of *Pythium*. Morphologically, it is an ornamented oogonia, like *P. oligandrum*.

### 3.2. Secretomes Contained Proteins, Amino Acids, and Phenolic Compounds

**Figure 2:**
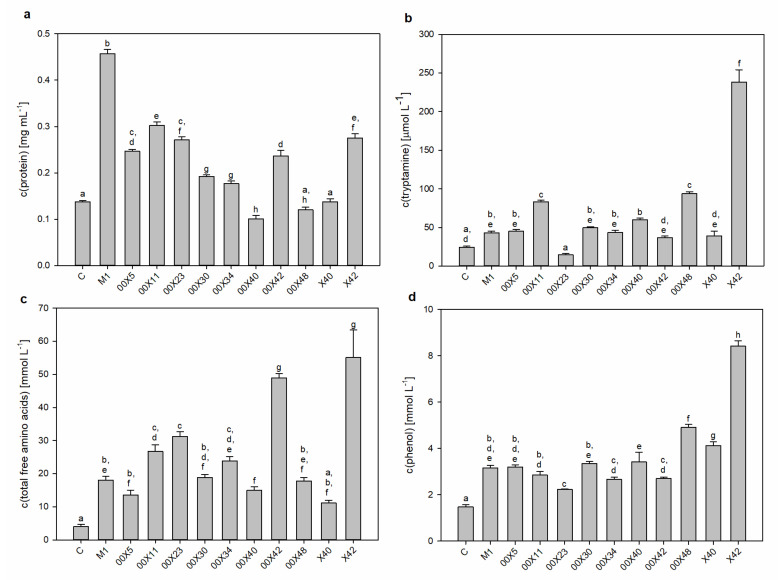
Characterization of compounds secreted into the medium by *Pythium* strains: protein content (**a**), tryptamine content (**b**), total free amino acids (**c**), total phenolics (**d**). Each code (00X5, 00X11, 00X23, 00X30, 00X34, 00X40, 00X42, 00X48, X40, X42, and M1) represents a *Pythium* strain. Control (C) is a sterile, non-inoculated cultivation medium. The different letters above each bar denote significant differences (*p* ≤ 0.05) between groups according to one-way ANOVA (Bonferroni method).

**Figure 3:**
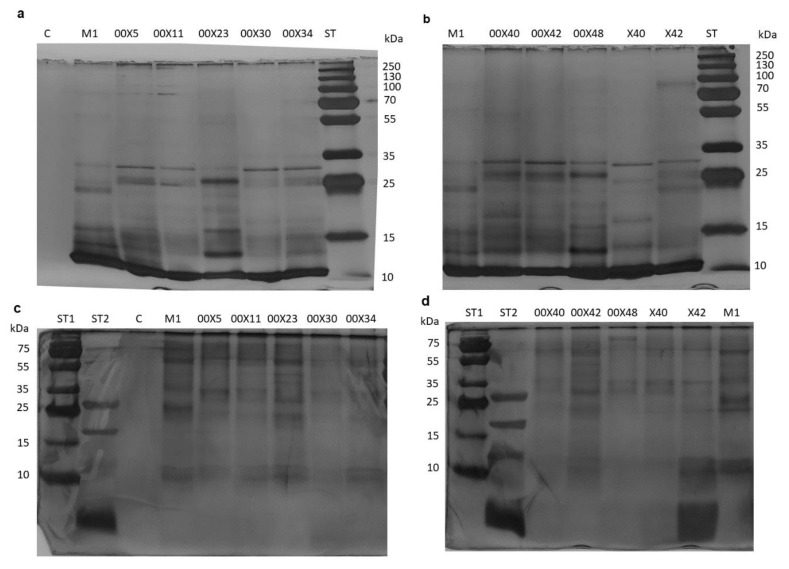
Electrophoretic separation of proteins secreted into the medium by *Pythium* strains. The proteins were separated by 15% SDS-PAGE (**a**,**b**) and 16% Tricine-SDS-PAGE (**c**,**d**). ST1-Thermo Scientific PageRuler Plus Prestained Protein Ladder (kDa): 250; 130; 100; 70; 55; 35; 25; 15; 10. ST2-Polypeptide SDS-PAGE Bio-Rad (kDa): 26.6; 17; 14.4; 6.5; 3.5; 1.4. Each code (00X5, 00X11, 00X23, 00X30, 00X34, 00X40, 00X42, 00X48, X42, and M1) represents rapeseed plants treated with a *Pythium* strain. Control (C) was prepared as untreated rapeseed plants.

**Figure 4:**
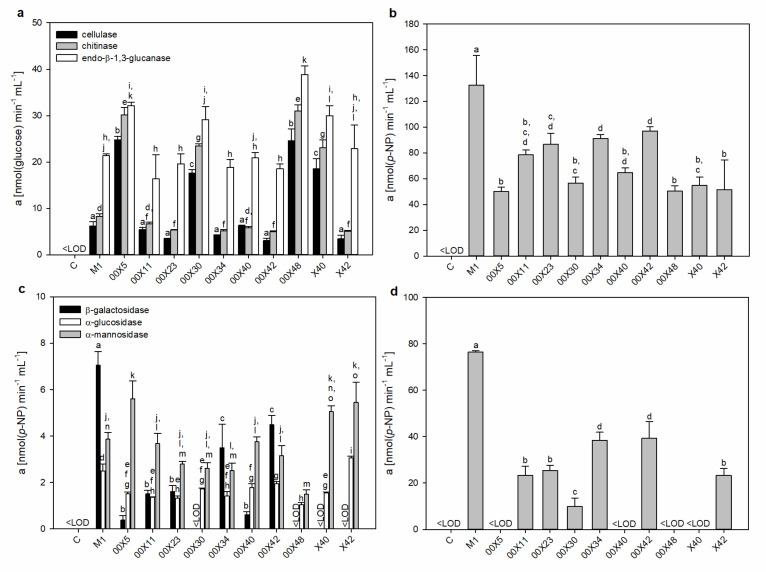
Activities of endo- and exoglycosidases secreted into the medium by *Pythium* strains. Cellulase, chitinase, and endo-β-1,3-glucanase activities (**a**); β-glucosidase activity (**b**); β-galactosidase, α-glucosidase, and α-mannosidase activities (**c**); β-galactosidase activity in alkaline pH 9.0 (**d**). Each code (00X5, 00X11, 00X23, 00X30, 00X34, 00X40, 00X42, 00X48, X40, X42, and M1) represents a *Pythium* strain. Control (C) is a sterile, non-inoculated cultivation medium. The different letters above each bar denote significant differences (*p* ≤ 0.05) between groups according to one-way ANOVA (Bonferroni method).

**Figure 5:**
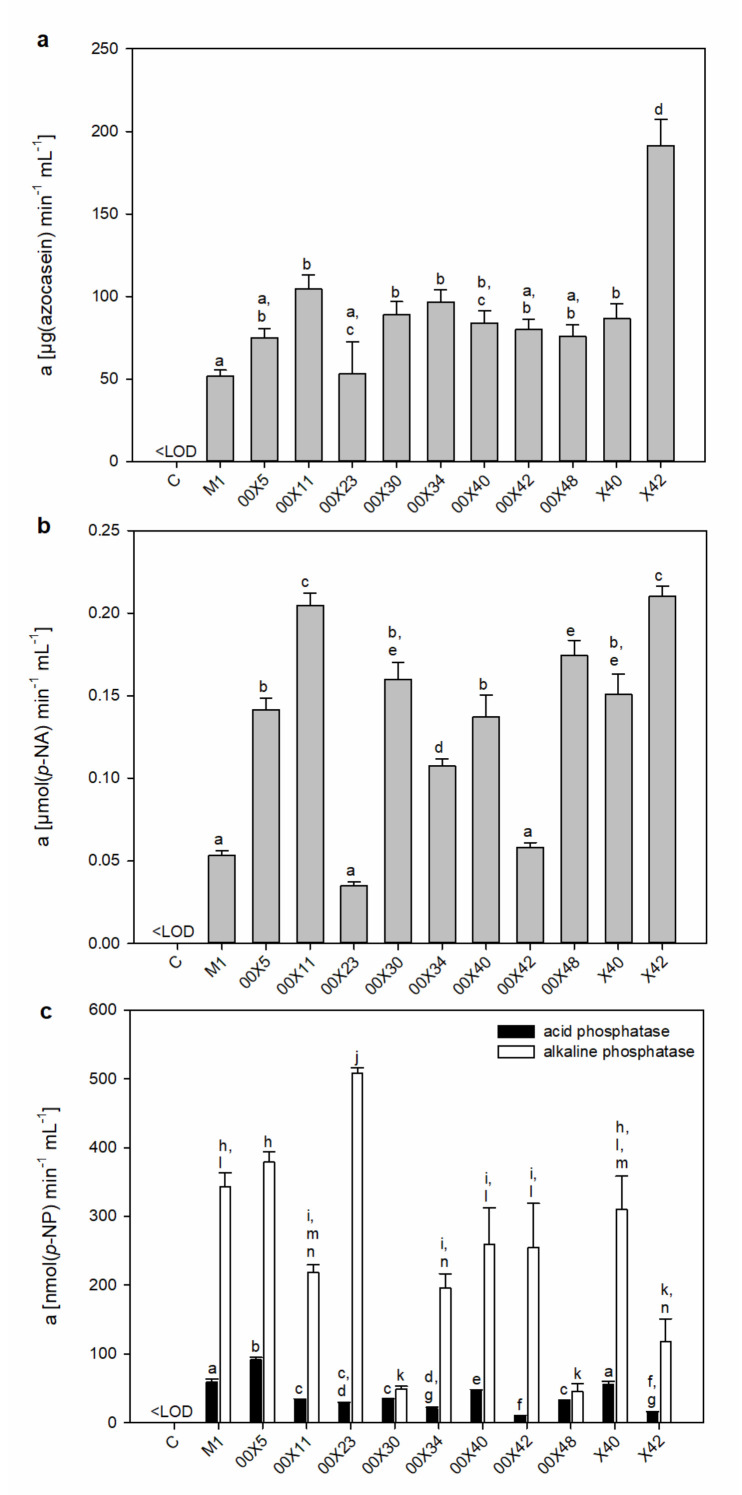
Activities of proteases and phosphatases secreted into the medium by *Pythium* strains. Total proteolytic activity (**a**), serine proteases (**b**), acid and alkaline phosphatases (**c**). Each code (00X5, 00X11, 00X23, 00X30, 00X34, 00X40, 00X42, 00X48, X40, X42, and M1) represents a *Pythium* strain. Control (C) is a sterile, non-inoculated cultivation medium. The different letters above each bar denote significant differences (*p* ≤ 0.05) between groups according to one-way ANOVA (Bonferroni method).

The main goal of this study was to find out the biochemical background of the *Pythium* spp. life strategies, i.e., mycoparasitism and/or promoting plant growth. The oomycetes depend on their hosts in acquiring β-hydroxy sterols for sporulation [6] and thus produce extracellular protein elicitors (oligandrin and cell wall protein fractions) such as sterol carriers. A side effect of these elicitors is plant priming and activation of plant defense responses, which is advantageous during interaction with real pathogens [1]. In addition, *Pythium* spp. as mycoparasites secrete a large arsenal of effector proteins that allows the penetration of the host cell wall [39] (Figure 2, Figure 3, Figure 4 and Figure 5). Although the composition of the medium was identical among all studied *Pythium* strains, the contents of proteins, amino acids, and phenolic compounds in secretomes varied considerably (Figure 2). All *Pythium* strains secreted proteins into the medium; the highest content was in M1 and the lowest in 00X40 (Figure 2a). Differences were also found in the proportion of individual proteins analyzed by SDS-electrophoresis (Figure 3). Most proteins were of a molecular weight lower than 30 kDa, but proteins were also detected in the regions around 85 kDa and 55 kDa. Strains 00X40 and X40, and strains 00X23 and 00X42 showed similar protein profiles. However, the protein profile of X42 was quite unique; the relative molecular weights of its most abundant proteins differed from those of other strains analyzed in this study. By Tricine electrophoresis, suitable for separating small proteins and peptides, we identified molecules of approximately 10 kDa, which could correspond to oligandrins. In addition to oligandrins, X42 medium contained several peptides (<6.5 kDa), which could be either elicitors or degradation products (Figure 3).

One of the significant properties of *P. oligandrum*, which is responsible for plant growth promotion, is the production of tryptamine, the precursor of the phytohormone auxin. Thus, we determined the concentrations of tryptophan and tryptamine in the medium (Table 2, Figure 2b). Although we found low tryptamine concentrations and no free tryptophan in the control medium (without *Pythium*), after *Pythium* cultivation, tryptophan was present and tryptamine concentrations were significantly elevated, especially in X42 (5.5 times more than M1). The lowest tryptamine concentration was found in 00X23 medium, whose value was only 6% of that in X42 (Table 2, Figure 2b). Considering the amount of tryptophan and tryptamine provided in their secretomes, strains X42, 00X42, and 00X11 appear to have good predispositions to increase plant growth and fitness (Table 2, Figure 2b).

Due to differences in tryptophan content, we also determined other free amino acids and total amino acids (Table 2, Figure 2c). In the control medium, tyrosine, glutamine, and aspartate were absent, whereas histidine, lysine, and arginine were found in very low concentrations (Table 2). Key amino acids that could affect plant metabolism (tryptophan, methionine, phenylalanine, and partly tyrosine) were most abundant in secretomes of *Pythium* spp. X42, 00X42, and 00X11 (Table 2). The only absent amino acid in all media was cysteine, which has a reactive thiol group and therefore is likely bound in other compounds such as glutathione. The concentration of all free amino acids increased in the medium after *Pythium* cultivation; in X42, the increase was 14 times in comparison with the control (Figure 2c). Although the specific secondary metabolites that *Pythium* spp. produce remain somewhat unclear, we tested for the presence of phenolic compounds (Figure 2d). The highest value was determined in strain X42, 2.7 times exceeding M1. The presence of phenylpropanoid compounds is unlikely because we did not find the initial enzyme catalyzing the conversion of phenylalanine to cinnamic acid, phenylalanine ammonium lyase, in the published genome of the *P. oligandrum* strain ATTC 38,472 [15]. However, the synthesis of isoprenoids is probable because in the abovementioned genome, we found genes for enzymes capable of synthesizing mevalonate, isopenthenyldiphosphate, geranyldiphosphate, farnesyldiphosphate, and geranylgeranyldiphosphate from an acetyl-CoA precursor. This is in agreement with other oomycetes [40].

### 3.3. Pythium Secretes a Cocktail of Host Cell Wall-Degrading Enzymes

Hydrolytic enzymes facilitate cell wall penetration for host colonization. These enzymes include cutinases, glycosidases, pectinases, and proteases. A “cocktail” of different hydrolytic enzymes with overlapping substrate specificities apparently enables the formation of proper infection structures [41]. Most information about these enzymes in oomycetes derives from genomic analysis of *Pythium* spp. [15,39]. The glycoside hydrolases and proteases were represented in high numbers. Among oomycete effector proteins, glycoside hydrolases belong to the most abundantly secreted proteins (79 for *P. oligandrum*). Based on sequence analysis, glycoside hydrolases can be assigned to families [39], but their true substrate specificity and activity cannot be ascertained. This is the first study providing a comprehensive survey of enzyme activities of plant and fungal cell wall-degrading enzymes across the variety of *Pythium* strains. We showed that there is a high difference in the quantity and quality of the presented enzymes, even across strains of the same species (Figure 4 and Figure 5), and proper strain selection is crucial in searching for BCAs.

In comparison with M1, higher activities of 12ellulose (3–4-fold), chitinase (3-fold), and endo-β-1,3-glucanase (1.5-fold) were found in 00X5, 00X30, 00X48, and X40 media (Figure 4a).

In addition to endoglycosidases, exoglycosidases also complete the degradation of glycoconjugates. Thus, we measured the activity of α-glucosidase, β-glucosidase, β-galactosidase, α-mannosidase (Figure 4), α-galactosidase, and β-hexosaminidase. β-Glucosidase degrading β-oligoglucans into glucose monomers, used as a source of energy, showed the highest activity, especially in M1 (Figure 4b). The other strains’ media reached only 40–70% of M1 activity. α-Glucosidase activity was approx. 45 times lower than β-glucosidase activity, as expected because substrates such as α-glucans are a much less commonly found source in the natural environment. α-Mannosidase may degrade mannan structures, glycoproteins, or glycolipids in host cell walls or membranes. The activity of α-mannosidase in 00X5 and X42 media exceeded M1 by 40–45%; and X40 by 30%. (Figure 4c). β-Galactosidase activity at pH 4.5 was found only in some *Pythium* spp. (Figure 4c). Interestingly, even though exoglycosidases generally have an acidic optimum pH, β-galactosidase showed activity when measured under alkaline reaction conditions; the highest activity at pH 4.5 was found in M1, with no activity in 00X30, 00X48, X40, or X42 (Figure 4c). This enzyme may have a very broad optimum pH, but the 00X30 and X42 media showed activity only under alkaline conditions, reaching only 10% and 30% of M1, respectively. Therefore, we cannot rule out an alkaline β-galactosidase isoform (Figure 4d). Various glycoconjugates containing galactose, hemicelluloses, and pectins in plant cell walls, glycolipids, and glycoproteins could be substrates of this enzyme. No activity of α-galactosidase or β-hexosaminidase was detected, but the activity of these enzymes could be induced by the presence of an appropriate substrate. As far as we know, this is one of the first studies concerning the determination of exoglycosidase activity in *Pythium* spp.

Numerous pathogenic fungi and oomycetes produce saponin-detoxifying glycosidases, which are able to deglycosylate saponin molecules for their detoxification (degradation) [42,43,44]. Another role of exoglucosidases, namely β-glucosidase, could be the re-activation (deglycosylation) of O-glycosylated cytokinins, thus modulating the phytohormonal levels in planta [45]. Glycosylation is also the key regulating factor of the phenylpropanoid pathway by changing the solubility, stability, and toxicity of secondary metabolites. A vast array of glycoside hydrolases could help oomycetes to alter host metabolism, especially the level of defense compounds [46].

In addition to glycosidase activity, proteolytic activity is important for the ability of *Pythium* to penetrate the cell wall of the host. Accordingly, total proteolytic activity was assessed using azocasein as a substrate. All *Pythium* strains secreted proteases into the medium, and the highest activity was found for X42, whereas the lowest activity was found for M1. The activity was almost 4 times higher in X42 than in M1 (Figure 5a). In addition to X42, 00X11 strains also showed high activity of serine proteases in the media (both ca. 4-fold higher than M1) (Figure 5b), with significant differences in the proportion of individual classes of proteases in *Pythium* spp.

Phosphatases are enzymes with broad substrate specificities. Both alkaline and acidic phosphatases were present in all *Pythium* spp. media (Figure 5c). Alkaline phosphatase activity was much higher than acidic phosphatase activity. The highest differences between them were found for 00X23 (19-fold) and 00X42 (26-fold). Phosphatases remove phosphate groups from small organic molecules or polymers, which are then used to meet their own needs. This feature could also be useful for increasing nutrient availability for plants when phosphate is released near the roots.

### 3.4. How Do Pythium spp. Affect Metabolism in Inoculated Rapeseed Plants?

The impact of inoculation of *Brassica napus* seeds with ten *Pythium* species on plant metabolism was followed in 3-week-old plants. Seed coating represents an efficient and convenient tool for introducing beneficial microorganisms into the soil and consequently into the rhizosphere or plant tissues [47]. Although most growth parameters were not affected in young plants, many metabolic pathways showed significant changes even within individual isolates. The inoculated plants showed no visible differences when compared to the control samples, or to each other. Similarly, the elementary analysis of dried leaf samples showed no significant differences in the content of carbon, nitrogen, or sulfur between plants treated with *Pythium* spp. and untreated control (Table S2). The carbon-to-nitrogen ratio, which characterizes the nitrogen supply to the plant, is shown in Figure S5. This ratio ranged from 6.2 to 8.2, and these differences were not significant, although the presence of various *Pythium* spp. may affect nitrogen use in plants. The content of photosynthetic pigments was also determined in leaf samples from experimental plants (Figure S6). The content of chlorophyll a was 11% and 28% higher in plants treated with M1 and 00X11 than in the control, respectively. The content of chlorophyll b was also higher in 00X11 and 00X42 than in other experimental groups. This corresponds to the previous assumption of positive influence of these strains on plant fitness.

**Figure 6:**
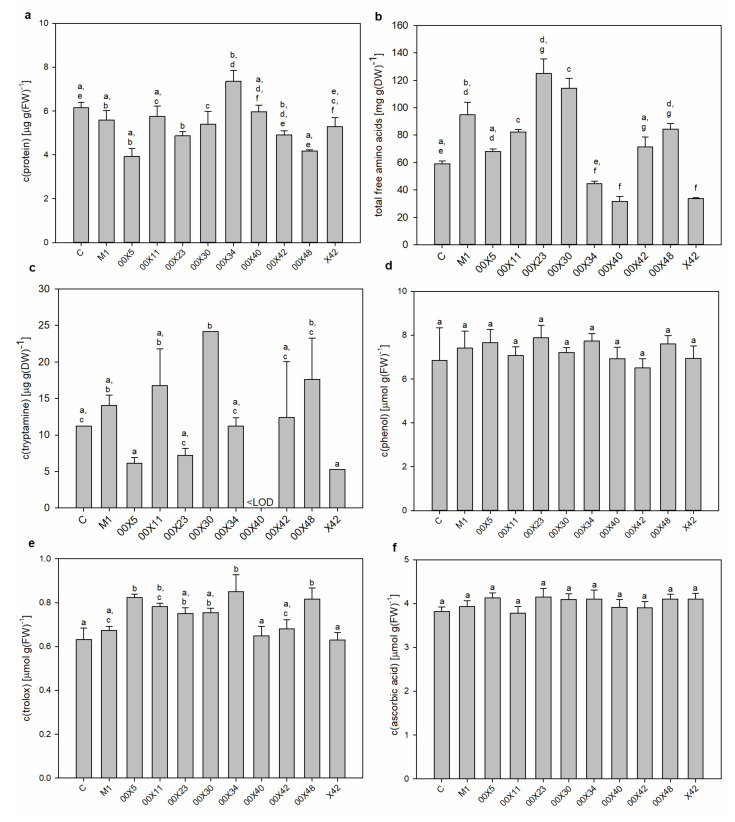
Characterization of the rapeseed leaves. Protein content (**a**), total free amino acids (**b**), tryptamine content (**c**), total phenolics (**d**), antioxidant capacity—2,2-diphenyl-1-picrylhydrazyl (DPPH) method (**e**), antioxidant capacity—Ferric Reducing Antioxidant Potential (FRAP) method (**f**). Each code (00X5, 00X11, 00X23, 00X30, 00X34, 00X40, 00X42, 00X48, X42, and M1) represents rapeseed plants treated with a *Pythium* strain. Control (C) was prepared as untreated rapeseed plants. The different letters above each bar denote significant differences (*p* ≤ 0.05) between groups according to one-way ANOVA (Bonferroni method). Abbreviations: DW, dry weight; FW, fresh weight.

**Figure 7:**
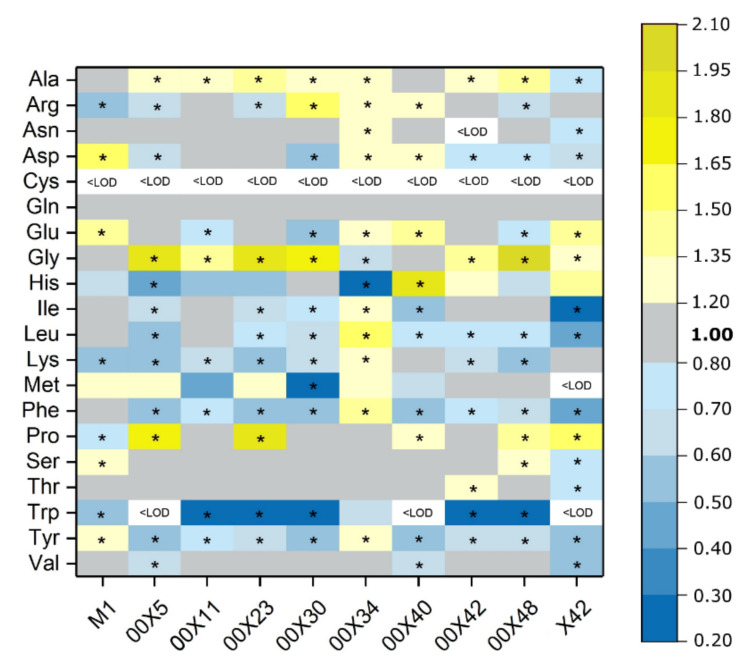
Free amino acid content in the rapeseed leaves. The contents of individual amino acids of all samples are expressed in % of total amount (dry weight) and compared with those of the control (untreated rapeseed plants). Each code (00X5, 00X11, 00X23, 00X30, 00X34, 00X40, 00X42, 00X48, X42, and M1) represents rapeseed plants treated with a *Pythium* strain. Asterisks denote significant differences (*p* ≤ 0.05) between sample and control plants according to *t*-test. <LOD, under the limit of detection.

In leaves, the soluble protein content varied, albeit not significantly (Figure 6a), and no differences in protein profile were detected by SDS-electrophoresis (Figure S7). However, some differences were found in the total amount of free amino acids. Plants inoculated with some *Pythium* strains showed increased total free amino acid content, whereas others showed lower content than the control (Figure 6b). More interestingly, the comparison of individual free amino acids (Figure 7) highlighted that the contribution of the first synthetized amino acid glutamine was equal in all plant groups. Conversely, the levels of some amino acids were decreased in all plant groups, e.g., aromatic amino acids, tryptophan, phenylalanine, and tyrosine. The decrease in tryptophan was particularly significant in plants treated with 00X40, 00X5, and X42 *Pythium* spp. Its concentration was under the detection limit; therefore, the tryptamine content was also measured (Figure 6c). However, tryptamine concentration was also low in plants treated with these strains, most likely because tryptamine was further metabolized to auxin. In turn, the plants inoculated with 00X30, 00X48, and 00X11 strains showed high tryptamine concentration, most likely due to slower conversion to auxin. The presence of tryptamine in control plants also documents its other uses and functions in plant metabolism. Alanine and glycine were among the more abundant free amino acids. The levels of proline, which is often synthetized in plants under stress conditions, were increased in some plant groups and unchanged or decreased in others (Figure 7).

Enhanced secondary metabolite synthesis, especially *via* phenylpropanoid and terpenoid pathways, has well-described effects on plant metabolism caused by *P. oligandrum* [3]. Surprisingly, no significant differences were found in phenolic content, although it was slightly elevated in some groups in comparison with the control (Figure 6d). The method detected only soluble phenolics and not compounds bound to the cell wall, so a significant part of polyphenols could have been incorporated as phenolic acids and especially lignin. Cell wall impregnation by lignin could significantly improve the defense against biotic stress. The soluble phenolic compounds contribute to the total antioxidative capacity; thus, this property was determined in plant extracts using two methods, DPPH (Figure 6e) and FRAP (Figure 6f). Considering the nonsignificant changes in phenolic content described above, the antioxidant capacity unsurprisingly showed no significant differences. Nevertheless, the FRAP method showed 20% enhancement of this value in plants treated with 00X5 and 00X34 strains.

### 3.5. The Concentration of Phytohormones is Affected by Pythium spp. in the Rapeseed Plants

**Figure 8:**
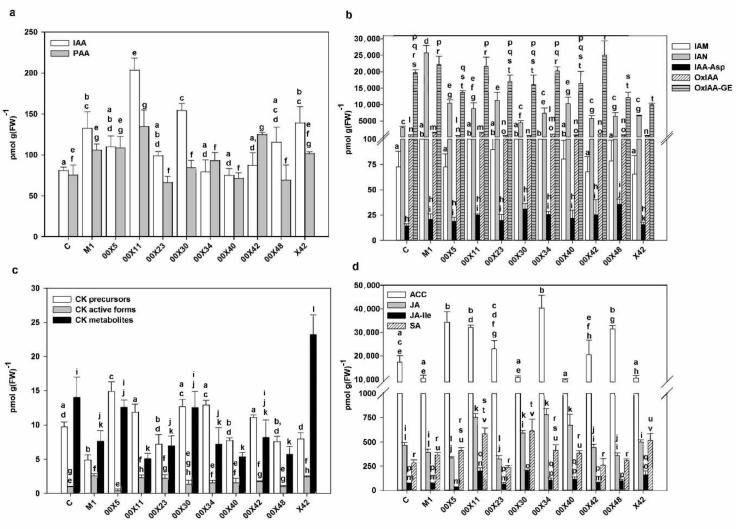
Phytohormone contents in the rapeseed leaves. Active auxins (**a**), precursors and deactivation products of auxins (**b**), cytokinins (**c**), aminocyclopropane-carboxylic acid, jasmonic acid; jasmonate-isoleucine, salicylic acid (**d**). Each code (00X5, 00X11, 00X23, 00X30, 00X34, 00X40, 00X42, 00X48, X42, and M1) represents rapeseed plants treated with a *Pythium* strain. Control (C) was prepared as untreated rapeseed plants. The different letters above each bar denote significant differences (*p* ≤ 0.05) between groups according to one-way ANOVA (Bonferroni method). Abbreviations: FW, fresh weight; ACC, 1-aminocyclopropane-1-carboxylic acid; CK, cytokinin; IAA, indole-3-acetic acid; IAA-Asp, IAA-aspartate; IAM, indole-3-acetamide; IAN, indole-3-acetonitrile; JA, jasmonic acid; JA-Ile, JA-isoleucine; OxIAA, oxo-IAA; OxIAA-GE, oxo-IAA-glucose ester; PAA, phenylacetic acid; SA, salicylic acid.

Plant growth and development as well as interactions with the environment are controlled by phytohormones. Accordingly, *P. oligandrum* may promote plant growth by increasing auxin concentrations. Indole-3-acetic acid (IAA), the most physiologically active auxin, may be synthetized from tryptamine provided by *P. oligandrum* [8]. Therefore, the auxin content of rapeseed plants treated with the *Pythium* strains was analyzed (Figure 8a). Considering the amount of tryptophan and tryptamine provided in their secretomes, the strains X42 and 00X11 appear to have good predispositions to increase plant growth and fitness (Table 2, Figure 2b). Indeed, the highest IAA content was found in plant leaves treated with 00X11, 00X30, X42, and M1 strains. Differences were also found in the contents of indole-3-acetamide (IAM) and indole-3-acetonitrile (IAN), which are precursors of IAA in different plant biosynthetic pathways (Figure 8b). In addition, differences were also found in the content of phenylacetic acid (PAA), a weak, evolutionarily ancient auxin. This compound was also present in higher amounts in some plants (e.g., treated with 00X11 or 00X42) than in the control (Figure 8a). These results suggest that *Pythium* likely affects auxin metabolism by multiple pathways other than just tryptamine delivery. The catabolic products of auxin metabolism were detected in all experimental groups (Figure 8b). Oxo-IAA and its glucosyl ester were the most abundant catabolites. Auxin inactivation is also performed by conjugation with amino acids, but this conjugation only occurred with aspartic acid in rapeseed plants. The highest contents of tryptamine (Figure 2c) and tryptophan (Table 2), determined in X42 medium, positively correlated with IAA level in leaves (Figure 8a). Negative correlation of tryptamine and tryptophan with the content of IAA degradation products (IAM, IAA-Asp, Ox-IAA, Ox-IAA GE) was not detected. The highest content of IAA and PAA was found in plants treated with 00X11 and 00X30 (Figure 8a).

The promotion of cell division and subsequently of plant growth requires cytokinins (CKs) as well as auxins. The active cytokinins, namely trans-zeatin and cis-zeatin, as well as their precursors, storage forms (CK O-glucosides), and degradation products (CK N-glucosides) were also determined in plants (Figure 8c). Higher concentrations of active cytokinins than in control (with the exceptions of 00X5 and 00X48) were found in all groups of rapeseed plants. High concentrations of precursors and degradation products in most experimental variants suggest intensive cytokinin metabolism.

In addition to auxins and cytokinins, hormones related to plant immunity were also monitored, particularly salicylic acid (SA) and jasmonate (JA) (Figure 8d). The treated rapeseed plants had higher SA content (except for 00X23 and 00X42) compared with the control. The highest SA concentrations were found in plants treated with 00X30 and 00X11, increasing to 216% and 205%, respectively. The contents of JA and especially of its active form JA-Ile were higher in plants treated with 00X11, 00X30, 00X34, and 00X40. SA and JA activate signaling pathways in response to biotroph or necrotroph/herbivore attack, respectively. Considering the possibility of attack by different pathogens, as well as the fact that some pathogens may change their lifestyle during infection, these hormones may enhance plant defense against a wide range of foes. The spatially separated activation of JA and SA defense pathways, which could increase the chance of successful elimination of greater pest diversity, has been described [48]. SA and JA functions are modulated by the presence of other phytohormones. Gaseous phytohormone ethylene (ET), the precursor of which is 1-aminocyclopropane-1-carboxylic acid (ACC), could modify the defense responses controlled by JA [49]. ACC was present in all leaf samples including untreated controls. Its concentration was in six groups higher than in the control; the highest increase was observed in plants treated with 00X34 (231%). The plants treated with the 00X11 strain contained higher concentrations of all aforementioned phytohormones (Figure 8, Table S1). The higher levels of JA, JA-Ile, and ACC in treated plants are in accordance with the reported induction of JA and ET signaling pathways by *P. oligandrum* elicitins [50,51]. In turn, SA signaling including PR1 and PR5 in tomato plants has been ruled out [50], but we found higher levels of SA in rapeseed plants inoculated with some *Pythium* spp. than in control (Figure 8d). However, it is possible to assume species specificity or simply only readiness to respond faster (and to a greater extent) in the case of infection. The concentrations of individual hormones and their metabolites are shown in Table S1.

### 3.6. Pythium Alters the Glucosinolate Composition of Inoculated Rapeseed Plants

Brassiacaceous plants contain glucosinolates, which protect their tissues from herbivores and likely also from pathogens. The profile of glucosinolates depends on the plant species, its genotype, and growth conditions including biotic and abiotic stresses [52]. Progoitrin and gluconapine were found to be the main glucosinolates in rapeseed meal [53]. In addition, glucoraphanin, glucobrassicanapin, and glucobrassicin were found in rapeseed plants [54]. In the leaves of our experimental plants, the most abundant glucosinolates were glucobrassicin and progoitrin. Furthermore, glucoraphanin was also identified, but only in plants treated with *Pythium* strains; in the control plants and in the plants grown with 00X34, the value was below the detection limit (Table 3). *Pythium* strains can alter glucosinolate content. In the *Pythium*-treated plants, glucobrassicin content was enhanced, whereas progoitrin was rather reduced. This could be advantageous, because progoitrin is metabolized to oxazolidine-2-thione, which causes reproductive and thyroid problems when consumed by animals [55,56]. In general, glucosinolates are of crucial importance in plant defense against herbivores [57] or plant pathogens [58,59,60]. Tryptophan is present in *Pythium*-treated plants at very low levels or under the detection limit (Figure 7); therefore, it could be a precursor not only of auxin but also of glucobrassicin (Table 3). Methionine as a precursor of glucoraphanin and progoitrin was secreted by *Pythium* strains into the medium, but its concentration in plants depended on the *Pythium* strain (Table 2, Figure 7). It is possible that methionine is provided by *Pythium* to the plant, thus stimulating metabolic pathways originating in methionine, e.g., biosynthesis of ethylene or S-adenosylmethionine, a methyl group donor [49]. It seems that seed coating could increase plant defense at a young growth stage when plants are most vulnerable and not ready for harvest. Each step towards higher natural plant resistance leads to a reduction in the use of pesticides.

### 3.7. Could Pythium spp. Support the Activity of Antioxidant Enzymes in Rapeseed Plants?

**Figure 9:**
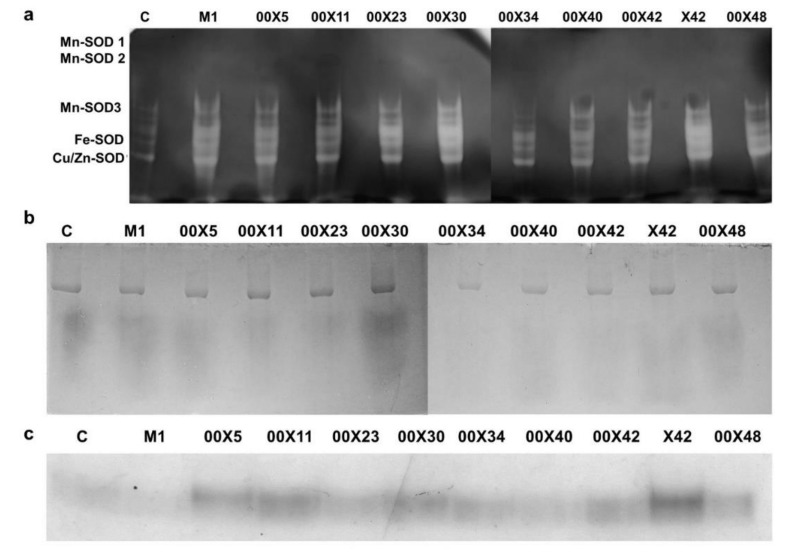
Detection of the activity of superoxide dismutase (SOD) isoenzymes (**a**), peroxidases (**b**), and shikimate dehydrogenase (**c**) after electrophoretic separation under native conditions. The individual SOD isoenzymes were determined in inhibition studies using H_2_O_2_ and KCN. Each code (00X5, 00X11, 00X23, 00X30, 00X34, 00X40, 00X42, 00X48, X42, and M1) represents rapeseed plants treated with a *Pythium* strain. Control (C) was prepared as untreated rapeseed plants.

The health status of a plant depends on the activity of antioxidant enzymes. The superoxide radical, one of the most dangerous reactive oxygen species, is detoxified by superoxide dismutase (SOD). SOD activity was detected in leaf extracts (Figure 9a). The individual SOD isoforms were more abundant in the plants inoculated with *Pythium* spp. than in the control plants; the highest intensity was found in 00X30 and X42 samples. The product of SOD reaction, H_2_O_2_, is also a reactive compound, which is degraded by catalases (CAT) (Figure S8a). CAT activity differed between plants; the highest activity was found in plants inoculated with X42, and the lowest in those treated with 00X11 and 00X42. H_2_O_2_ is also a substrate for peroxidases, which have broad substrate specificity. They participate in the synthesis of lignin, for example. At least two peroxidase isoforms were present. This microheterogeneity of the latter isoform could have been caused by glycosylation (Figure 9b). Another enzyme that uses H_2_O_2_ as a substrate is ascorbate peroxidase (Figure S8b). Its activity was higher in plants treated with *Pythium* spp. than in controls, except for 00X23 and 00X48.

Glutathione in its reduced form is important for the control of the reduced status of thiol groups in many proteins in plant cells. The change of glutathione from the oxidized form to the reduced one is catalyzed by glutathione reductase (GR), which uses NADPH as a coenzyme. However, the activity of GR in plants was not significantly affected by the *Pythium* spp. treatment (Figure S8c).

Plant redox state is also affected by the availability of NADPH, which is produced in chloroplasts during photosynthesis. The requirement for NADPH is enhanced under oxidative stress, and therefore other sources, such as NADPH-producing enzymes, could be used. However, the activity of the NADP-malic enzyme (NADP-ME) and of glucose-6-phosphate dehydrogenase (G6PDH) in experimental plants showed no significant effects from *Pythium* spp. The highest activity of NADP-ME and G6PDH was in plants treated with 00X48 and 00X34, respectively (Figure S8d,e).

NADPH is used also by enzymes of the shikimate pathway, which synthetize aromatic amino acids. The shikimate dehydrogenase (SDH) activity was higher in plants inoculated with *Pythium* spp. compared to the control, and the highest activity was found in plants treated with X42 (Figure 9c). The synthesis of aromatic amino acids, especially phenylalanine and partly tyrosine, is important for plants, not only for protein synthesis but also for the production of phenylpropanoid compounds including lignin, which fortifies cell walls and thus improves plant defense.

## 4. Conclusions

In this study, we assess a broad number of strains for traits related to mycoparasitism as well as plant-promoting activity. The secretomes of *Pythium* spp. varied significantly in terms of the activities of enzymes capable of acting on host cell walls. Even closely related *P. oligandrum* strains differed in their properties and effects on plants. The alterations in enzyme activities and tryptamine production may indicate advantages either for mycoparasitism or plant growth promotion. Some strains showed equal prerequisites for both effects. The preferred life strategy of *Pythium* spp. has a crucial importance in the selection of new biocontrol agents. This is the first study screening seed coating with *Pythium* spp. for effects on the metabolism of rapeseed plants. In addition to differences in free amino acids and antioxidant enzyme activities, major differences in phytohormone levels were also found, showing increased concentrations of auxin and jasmonates (and salicylic acid in some strains), indicating plant growth promotion as well as readiness for effective defense.

## Figures and Tables

**Table 1:** Summary of *Pythium* spp. and their identification parameters (internal transcribed spacer (ITS) rDNA and cytochrome c oxidase subunit I (COI)).

Identity	Strain	Accession No.	
		ITS rDNA	COI
*P. oligandrum*	00X5	*LR760206*	*LR760205*
*P. oligandrum*	00X11	*MT249384*	*LR760204*
*P. oligandrum*	00X23		*LR760202*
*P. oligandrum*	00X30		*LR760200*
*P. oligandrum*	00X34		*LR760199*
*P. oligandrum*	00X40	*LR760209*	*LR760201*
*P. oligandrum*	00X42	*LR760210*	*LR760197*
*P. oligandrum*	00X48	*LR760208*	*LR760198*
*P. oligandrum*	X40	*LR760207*	*LR760196*
*Pythium* sp.	X42	*LR760211*	*LR760203*

**Table 2:** The free amino acid content in the medium. <LOD, under the limit of detection. Each code (00X5, 00X11, 00X23, 00X30, 00X34, 00X40, 00X42, 00X48, X40, X42, and M1) represents a *Pythium* strain. Control (C) is a sterile, non-inoculated cultivation medium.

	C [µmol.L^-1^]											
	C	M1	00X5	00X11	00X23	00X30	00X34	00X40	00X42	00X48	X40	X42
Ala	993 ± 70	2578 ± 134	1523 ± 120	3055 ± 199	7391 ± 198	1951 ± 52	2984 ± 61	1625 ± 32	9734 ± 6	1832 ± 37	1130 ± 45	8088 ± 1769
Arg	32 ± 8	975 ± 50	1274 ± 42	1507 ± 66	457 ± 25	1272 ± 21	1036 ± 41	820 ± 8	908 ± 59	2039 ± 89	1057 ± 83	1088 ± 89
Asn	345 ± 39	685 ± 97	482 ± 109	1167 ± 80	784 ± 14	812 ± 43	1056 ± 65	794 ± 19	1171 ± 54	623 ± 92	442 ± 28	1462 ± 183
Asp	<LOD	426 ± 33	181 ± 50	1077 ± 219	709 ± 157	353 ± 130	854 ± 85	455 ± 98	2027 ± 67	295 ± 113	225 ± 131	2552 ± 120
Cys	<LOD	<LOD	<LOD	<LOD	<LOD	<LOD	<LOD	<LOD	<LOD	<LOD	<LOD	<LOD
Gln	<LOD	1207 ± 24	771 ± 12	2555 ± 241	2486 ± 65	1488 ± 116	2410 ± 102	2169 ± 115	3609 ± 94	777 ± 36	797 ± 47	4169 ± 630
Glu	63 ± 31	1731 ± 59	1208 ± 112	2737 ± 132	3332 ± 90	1934 ± 7	2806 ± 87	1415 ± 79	7537 ± 106	1106 ± 30	991 ± 30	8597 ± 937
Gly	276 ± 31	1223 ± 60	476 ± 69	1638 ± 137	2445 ± 107	861 ± 69	1726 ± 28	806 ± 17	4364 ± 181	778 ± 43	417 ± 83	4338 ± 927
His	1 ± 0	251 ± 13	204 ± 35	383 ± 37	128 ± 8	249 ± 1	337 ± 23	199 ± 27	351 ± 8	250 ± 46	163 ± 14	360 ± 111
Ile	106 ± 64	650 ± 71	471 ± 81	1038 ± 48	705 ± 45	645 ± 15	901 ± 87	517 ± 19	1365 ± 70	533 ± 47	371 ± 12	1662 ± 147
Leu	659 ± 43	1237 ± 12	1068 ± 161	1895 ± 67	1367 ± 109	1311 ± 52	1602 ± 87	1014 ± 132	2513 ± 66	1193 ± 92	811 ± 121	3099 ± 514
Lys	26 ± 7	900 ± 113	750 ± 26	1327 ± 32	779 ± 53	935 ± 20	1096 ± 17	679 ± 11	1408 ± 55	896 ± 27	546 ± 19	1669 ± 100
Met	134 ± 28	352 ± 10	323 ± 45	498 ± 147	313 ± 47	386 ± 31	331 ± 84	256 ± 27	640 ± 55	316 ± 29	251 ± 14	737 ± 129
Phe	138 ± 67	521 ± 47	374 ± 84	904 ± 182	476 ± 127	534 ± 43	748 ± 69	460 ± 116	910 ± 82	510 ± 38	354 ± 19	1441 ± 240
Pro	573 ± 146	2030 ± 155	2080 ± 292	1970 ± 163	4881 ± 89	2866 ± 115	1494 ± 105	1199 ± 102	3888 ± 108	3829 ± 138	1677 ± 89	6118 ± 983
Ser	228 ± 45	879 ± 22	558 ± 99	1331 ± 67	1865 ± 102	809 ± 44	1249 ± 78	734 ± 79	2859 ± 77	715 ± 133	470 ± 12	3113 ± 393
Thr	170 ± 42	846 ± 0	520 ± 63	1211 ± 102	1358 ± 47	793 ± 35	1116 ± 125	633 ± 28	2068 ± 42	712 ± 43	420 ± 22	2417 ± 452
Trp	<LOD	122 ± 12	50 ± 15	141 ± 56	87 ± 56	89 ± 17	127 ± 24	81 ± 14	266 ± 46	82 ± 21	60 ± 20	250 ± 61
Tyr	<LOD	419 ± 65	378 ± 78	676 ± 26	339 ± 26	440 ± 34	555 ± 58	351 ± 55	842 ± 32	376 ± 31	277 ± 32	1118 ± 238
Val	302 ± 31	1058 ± 158	821 ± 35	1623 ± 47	1353 ± 88	1095 ± 11	1443 ± 102	784 ± 113	2461 ± 71	884 ± 41	656 ± 41	2806 ± 347

**Table 3:** The contents of glucosinolates in the rapeseed leaves. Each code (00X5, 00X11, 00X23, 00X30, 00X34, 00X40, 00X42, 00X48, X42, and M1) represents rapeseed plants treated with a *Pythium* strain. Control (C) was prepared as untreated rapeseed plants. The different letters in upper index denote significant differences (*p* ≤ 0.05) between groups according to one-way ANOVA (Bonferroni method). Abbreviations: DW, dry weight.

	Glucobrassicin	Glucoraphanin	Progoitrin
	[mg g(DW)^−1^]	[mg g(DW)^−1^]	[mg g(DW)^−1^]
C	0.142 ± 0.002 ^a^	0 ± 0 ^i^	0.196 ± 0.004 ^l,n^
M1	0.214 ± 0.004 ^b,c^	0.001 ± 0.000 ^i^	0.133 ± 0.008 ^m,p^
00X5	0.200 ± 0.004 ^b^	0.002 ± 0.002 ^i,j^	0.166 ± 0.005 ^l,m^
00X11	0.230 ± 0.008 ^c^	0.003 ± 0.000 ^i,j^	0.139 ± 0.004 ^m,p^
00X23	0.180 ± 0.003 ^d^	0.009 ± 0.004 ^j,k^	0.210 ± 0.007 ^n,q^
00X30	0.418 ± 0.013 ^e^	0.005 ± 0.004 ^i,j^	0.142 ± 0.026 ^m,p^
00X34	0.049 ± 0.005 ^f^	0.014 ± 0.004 ^k^	0.086 ± 0.022 ^o^
00X40	0.097 ± 0.002 ^g^	0.005 ± 0.001 ^i,j^	0.120 ± 0.005 ^o,p^
00X42	0.318 ± 0.003 ^h^	0.003 ± 0.001 ^i,j^	0.226 ± 0.018 ^n^
00X48	0.179 ± 0.002 ^d^	0.005 ± 0.002 ^i,j^	0.175 ± 0.008 ^l,m,q^
X42	0.148 ± 0.003 ^a^	0 ± 0 ^i^	0.078 ± 0.017 ^o^

## Supplementary Materials

The following are available online at https://www.mdpi.com/2076-2607/8/10/1472/s1. Figure S1. The in vitro interaction between *Pythium oligandrum*—strain M1 and *Alternaria alternate*, Figure S2. The in vitro interaction between *Pythium oligandrum*—strain M1 and *Verticillium albo-atrum*, Figure S3. The in vitro interaction between *Pythium oligandrum*—strain M1 and *Alternaria brasicae,* Figure S4. The in vitro interaction between *Pythium oligandrum*—strain M1 and *Phoma lingam,* Figure S5. C/N ratio in the rapeseed leaves, Figure S6. Chlorophyll and carotenoid content in the rapeseed leaves, Figure S7. Proteins of the rapeseed leaves separated by SDS-electrophoresis, Figure S8. Enzyme activities in the rapeseed leaves: catalase (a), ascorbate peroxidase (b), glutathione reductase (c), malic enzyme (d), glucose-6-phosphate dehydrogenase (e). The different letters above each bar denote significant differences (*p* ≤ 0.05) among the groups according to one way-ANOVA. Each code (00X5, 00X11, 00X23, 00X30, 00X34, 00X40, 00X42, 00X48, X42, and M1) represents rapeseed plants treated with *Pythium* strain. Control (C) was prepared as untreated rapeseed plants. Abreviations: FW, fresh weight, Table S1, Table S2. Carbon, nitrogen, sulfur, and hydrogen content in the rapeseed leaves, expressed as % of dry weight
