# Supplementary material for: Novel Insights into the Effect of Pythium Strains on Rapeseed Metabolism
Source: Microorganisms. 2020 Sep 25;8(10):1472. doi: 10.3390/microorganisms8101472 (PMC7650609; doi:10.3390/microorganisms8101472)
Supplement: Supplementary file 1 [file microorganisms-08-01472-s001.zip › microorganisms-922189-suppl.docx]

**SUPPLEMENT**

Novel Insights into the Effect of *Pythium* Strains on Rapeseed Metabolism

Kateřina Bělonožníková ^1^, Kateřina Vaverová ^1^, Tomáš Vaněk ^2^, Miroslav Kolařík ^3,4^,
Veronika Hýsková ^1^, Radomíra Vaňková ^5^, Petre Dobrev ^5^, Tomáš Křížek ^6^, Ondřej Hodek ^6^, Kateřina Čokrtová ^6^, Adam Štípek ^2^ and Helena Ryšlavá ^1,^*

^1^ Department of Biochemistry, Faculty of Science, Charles University, Hlavova 2030, 128 43 Prague 2,
Czech Republic; katerina.belonoznikova@natur.cuni.cz (K.B.); katerina.vaverova@natur.cuni.cz (K.V.); veronika.hyskova@natur.cuni.cz (V.H.)

^2^ Biopreparáty, spol. s r.o., Tylišovská 1, 160 00 Prague 6, Czech Republic; vanek@biopreparaty.eu (T.V.); stipek@biopreparaty.eu (A.Š.)

^3^ Institute of Microbiology, Academy of Sciences of the Czech Republic, Vídeňská 1083, 142 20 Prague 4, Czech Republic; mkolarik@biomed.cas.cz

^4^ Department of Botany, Faculty of Science, Charles University, Benátská 2, 128 01 Prague 2, Czech Republic

^5^ Institute of Experimental Botany, Academy of Sciences of the Czech Republic, Rozvojová 263, 165 02 Prague 6, Czech Republic; Vankova@ueb.cas.cz (R.V.); Dobrev@ueb.cas.cz (P.D.)

^6^ Department of Analytical chemistry, Faculty of Science, Charles University, Hlavova 2030, 128 43 Prague 2, Czech Republic; tomas.krizek@natur.cuni.cz (T.K.); ondrej.hodek@natur.cuni.cz (O.H.); katerina.cokrtova@natur.cuni.cz (K.Č.)

***** Correspondence: [helena.ryslava@natur.cuni.cz](mailto:helena.ryslava@natur.cuni.cz)

*This paper is dedicated to the memory of our inspirational colleague, teacher and friend, Prof. Marie Stiborová, who recently passed away.*


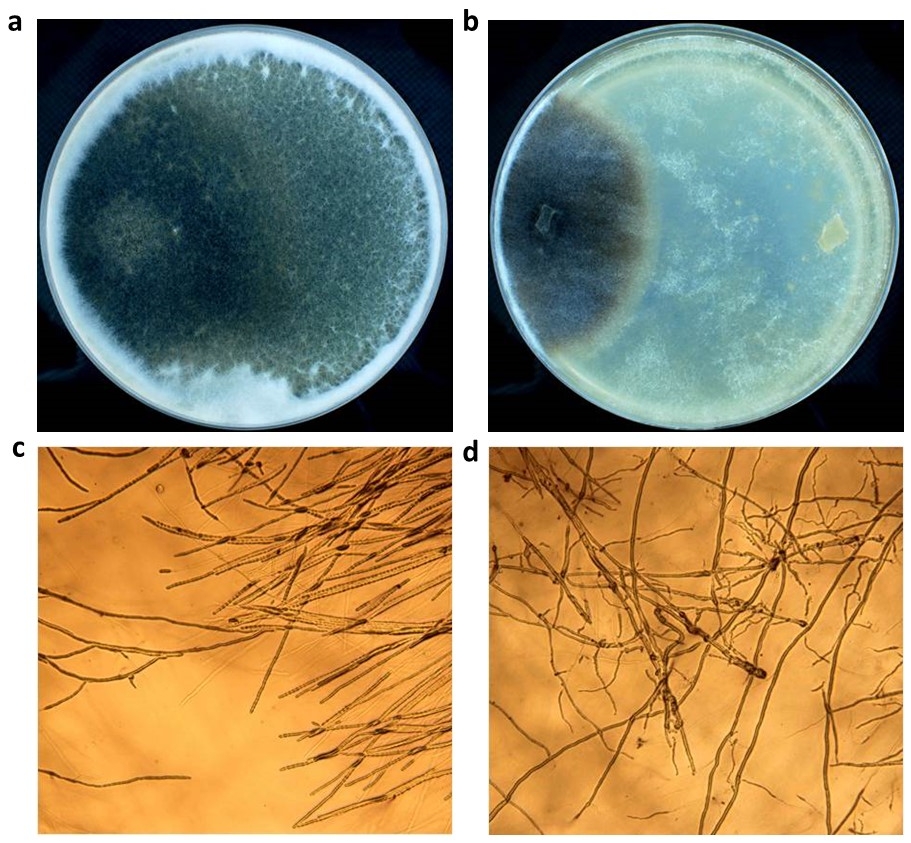


**Figure S1. The *in vitro* interaction between *Pythium* *oligandrum* – strain M1 and *Alternaria alternata*.** The control growth of *A. alternata* colonies (a, c). The parasitism effect of *P. oligandrum* – strain M1 was evaluated as overgrowth of the pathogen colony by *Pythium* sp. hyphae, cessation of pathogen hyphae growth loss or inability of the pathogen colony to change pigmentation in comparison with the control (b). The ability of *P. oligandrum* - strain M1 hyphae to encircle the pathogen hyphae at sites of early contact between the two colonies was observed (d).


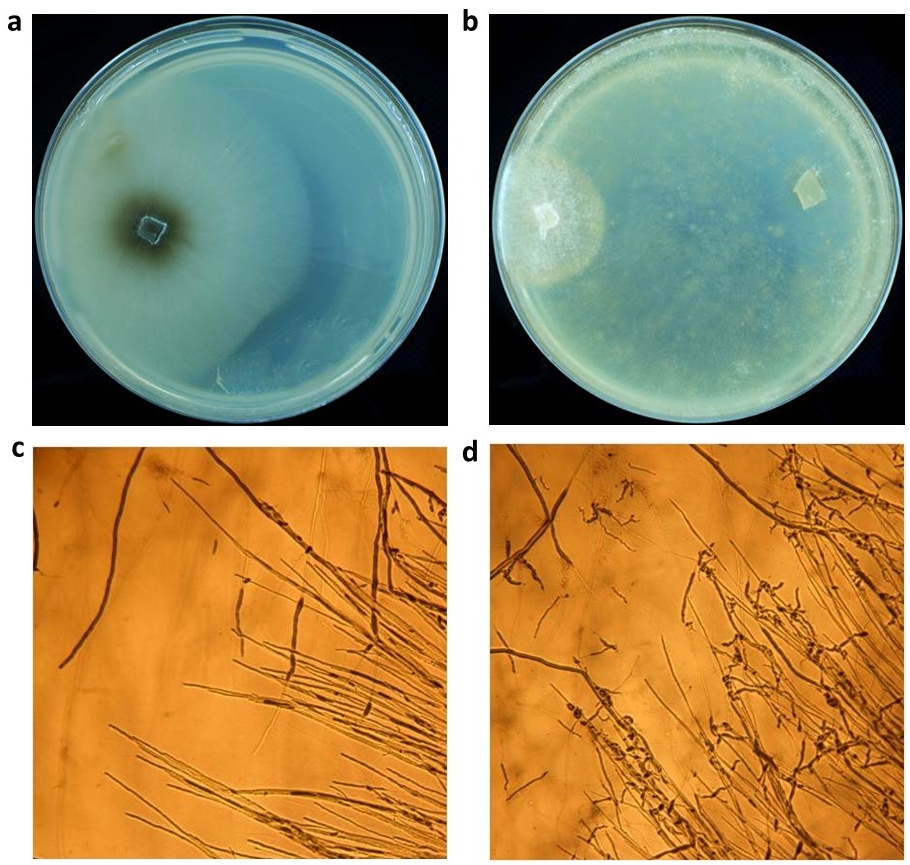


**Figure S2. The *in vitro* interaction between *Pythium* *oligandrum* – strain M1 and *Verticillium albo-atrum*.** The control growth of *V. albo-atrum* colonies (a, c). The parasitism effect of *P. oligandrum* – strain M1 was evaluated as overgrowth of the pathogen colony by *Pythium* sp. hyphae, cessation of pathogen hyphae growth loss or inability of the pathogen colony to change pigmentation in comparison with the control (b). The ability of *P. oligandrum* - strain M1 hyphae to encircle the pathogen hyphae at sites of early contact between the two colonies was observed (d).


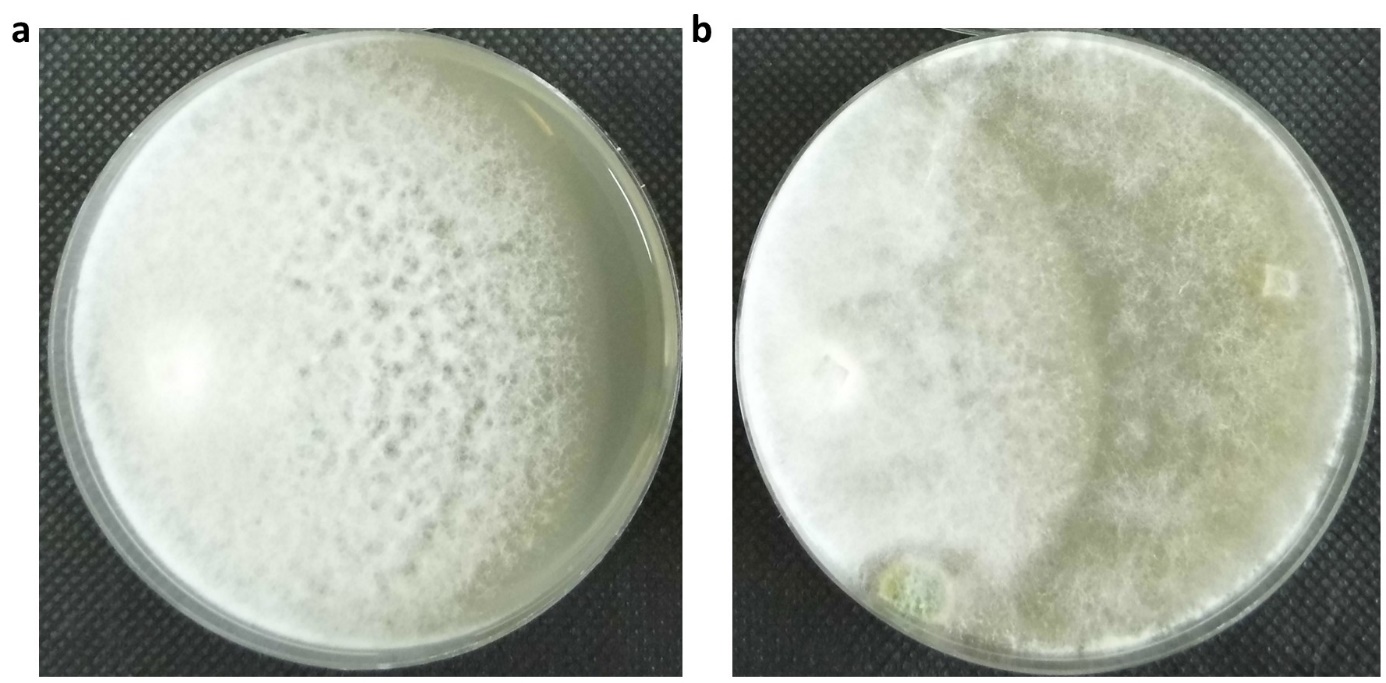


**Figure S3. The *in vitro* interaction between *Pythium* *oligandrum* – strain M1 and *Alternaria brasicae*.** The control growth of *A. brasicae* colonies (a). The parasitism effect of *P. oligandrum* – strain M1 was evaluated as overgrowth of the pathogen colony by *Pythium* sp. hyphae, cessation of pathogen hyphae growth loss or inability of the pathogen colony to change pigmentation in comparison with the control (b).


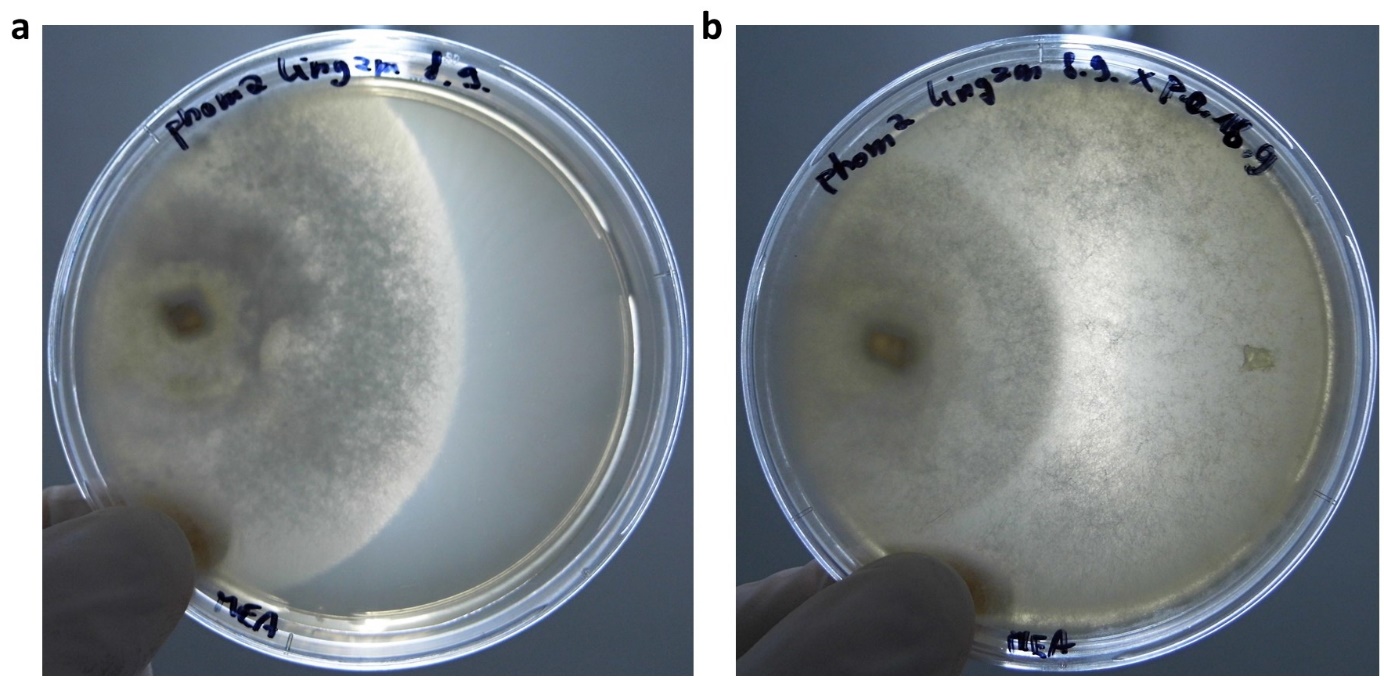


**Figure S4. The *in vitro* interaction between *Pythium* *oligandrum* – strain M1 and *Phoma lingam*.** The control growth of *P. lingam* colonies (a). The parasitism effect of *P. oligandrum* – strain M1 was evaluated as overgrowth of the pathogen colony by *Pythium* sp. hyphae, cessation of pathogen hyphae growth loss or inability of the pathogen colony to change pigmentation in comparison with the control (b).





**Figure S5. C/N ratio in the rapeseed leaves.** The different letters above each bar denote significant differences (P≤0.05) between groups according to one way-ANOVA. The same letters above a bar indicate no statistically significant differences. Each code (00X5, 00X11, 00X23, 00X30, 00X34, 00X40, 00X42, 00X48, X42, and M1) represents rapeseed plants treated with *Pythium* strain. Control (C) was prepared as untreated rapeseed plants.





**Figure S6. Chlorophyll and carotenoid content in the rapeseed leaves.** The different letters above each bar denote significant differences (P≤0.05) between groups according to one way-ANOVA. Each code (00X5, 00X11, 00X23, 00X30, 00X34, 00X40, 00X42, 00X48, X42, and M1) represents rapeseed plants treated with *Pythium* strain. Control (C) was prepared as untreated rapeseed plants.


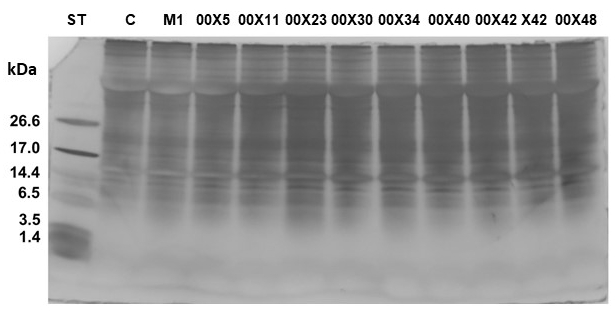


**Figure S7. Proteins of the rapeseed leaves separated by SDS-electrophoresis.** Abbreviations: ST – protein standard. Each code (00X5, 00X11, 00X23, 00X30, 00X34, 00X40, 00X42, 00X48, X42, and M1) represents rapeseed plants treated with *Pythium* strain. Control (C) was prepared as untreated rapeseed plants.





**Figure S8.** Enzyme activities in the rapeseed leaves: catalase (a), ascorbate peroxidase (b), glutathione reductase (c), malic enzyme (d), glucose-6-phosphate dehydrogenase (e). The different letters above each bar denote significant differences (P≤0.05) among the groups according to one way-ANOVA. Each code (00X5, 00X11, 00X23, 00X30, 00X34, 00X40, 00X42, 00X48, X42, and M1) represents rapeseed plants treated with *Pythium* strain. Control (C) was prepared as untreated rapeseed plants. Abreviations: FW, fresh weight

**Table S2. Carbon, nitrogen, sulfur, and hydrogen content in the rapeseed leaves, expressed as % of dry weight.** Each code (00X5, 00X11, 00X23, 00X30, 00X34, 00X40, 00X42, 00X48, X42, and M1) represents rapeseed plants treated with *Pythium* strain. Control (C) was prepared as untreated rapeseed plants.

|  | **N [%]** | **C [%]** | **H [%]** | **S [%]** | **C/N [%]** |
| --- | --- | --- | --- | --- | --- |
| **C** | 6.5±0.4 | 40.9±0.4 | 5.9±0.1 | 0.03±0.01 | 6.3±0.4 |
| **M1** | 5.6±0.2 | 40.0±0.2 | 6.0±0.0 | 0.05±0.03 | 7.1±0.2 |
| **00X5** | 5.3±0.3 | 40.5±0.7 | 6.0±0.1 | 0.06±0.06 | 7.7±0.3 |
| **00X11** | 6.5±0.1 | 39.9±0.2 | 5.9±0.1 | 0.06±0.04 | 6.2±0.0 |
| **00X23** | 5.0±0.1 | 41.4±0.4 | 6.1±0.1 | 0.07±0.05 | 8.2±0.2 |
| **00X30** | 6.3±0.6 | 39.9±0.1 | 6.4±0.7 | 0.09±0.05 | 6.5±0.7 |
| **00X34** | 6.0±0.1 | 39.4±0.9 | 5.8±0.2 | 0.05±0.03 | 6.5±0.1 |
| **00X40** | 5.6±0.2 | 40.8±0.4 | 6.0±0.1 | 0.05±0.03 | 7.3±0.2 |
| **00X42** | 5.9±0.1 | 41.1±0.1 | 6.1±0.0 | 0.05±0.03 | 7.0±0.1 |
| **00X48** | 5.2±0.1 | 41.1±0.4 | 6.0±0.2 | 0.07±0.04 | 7.8±0.3 |
| **X42** | 5.8±0.6 | 40.1±0.1 | 7.2±0.9 | 0.06±0.03 | 7.0±0.8 |
